# The World Starts With Me: A multilevel evaluation of a comprehensive sex education programme targeting adolescents in Uganda

**DOI:** 10.1186/1471-2458-11-334

**Published:** 2011-05-18

**Authors:** Liesbeth E Rijsdijk, Arjan ER Bos, Robert AC Ruiter, Joanne N Leerlooijer, Billie de Haas, Herman P Schaalma

**Affiliations:** 1 Windesheim University of Applied Sciences, Windesheim Honours College, Koestraat 3, 8011 NB Zwolle, The Netherlands; 2 Maastricht University, Department of Work and Social Psychology, the Netherlands; 3 Rutgers WPF, Utrecht, the Netherlands; 4 Groningen University, the Netherlands

## Abstract

**Background:**

This paper evaluates the effect of the World Starts With Me (WSWM), a comprehensive sex education programme in secondary schools in Uganda. The aim of the present study was to assess the effects of WSWM on socio-cognitive determinants of safe sex behaviour (delay; condom use and non-coercive sex).

**Methods:**

A survey was conducted both before and immediately after the intervention among students in intervention (*N *= 853) and comparison (*N *= 1011) groups. A mixed model repeated measures analysis was performed to assess the effectiveness of the WSWM programme on the main socio-cognitive determinants of safe sex behaviour at post-test. A similar post-hoc comparison was made between schools based on completeness and fidelity of implementation of WSWM.

**Results:**

Significant positive effects of WSMW were found on beliefs regarding what could or could not prevent pregnancy, the perceived social norm towards delaying sexual intercourse, and the intention to delay sexual intercourse. Furthermore, significant positive effects of WSWM were found on attitudes, self-efficacy and intention towards condom use and on self-efficacy in dealing with sexual violence (pressure and force for unwanted sex). A reversed effect of intervention was found on knowledge scores relating to non-causes of HIV (petting, fondling and deep kissing). A follow-up comparison between intervention schools based on completeness of the programme implementation revealed that almost all significant positive effects disappeared for those schools that only implemented up to 7 out of 14 lessons. Another follow-up analysis on the basis of implementation fidelity showed that schools with a "partial" fidelity score yielded more significant positive effects than schools with a "full" fidelity of implementation score.

**Conclusions:**

The study showed an intervention effect on a number of socio-cognitive determinants. However, the effectiveness of WSWM could be improved by giving more systematic attention to the context in which such a programme is to be implemented. Implications for the systematic development and implementation of school-based safe sex interventions in Uganda will be discussed.

## Background

In many Sub-Saharan countries, but also elsewhere, unsafe sexual behaviour among adolescents is widespread, as reflected by an early age onset of sexual intercourse, a large proportion of unprotected sexual intercourse, and multiple sexual partners among those who are sexually active [1-5]. In addition, coerced sexual intercourse among young people is a major problem in Sub-Saharan countries [3-5]. Uganda, being one of the worlds' poorest countries and having the youngest population in the world with a median age of 15, is no exception [5].

Although Ugandan adolescents are now delaying (first) sexual intercourse more than before [6], the majority have become sexually active by the age of 18 [3,7]. Ugandan girls start sexual intercourse at a younger age than boys: 15.5% of the girls have had sexual intercourse before the age of 15, rising to 64.2% by the age of 18, as compared to 12.2% and 49.9% of the boys [3,6]. When sexually active, only around 15% of the young Ugandans aged 15 to 19 years have ever used a condom. Of those aged 15-24 years who have ever had sex, 28% used a condom the first time that they had sex, with a slight difference between boys (29%) and girls (27%) [3].

Until the end of 2000, HIV prevalence in Uganda was the highest among adolescents (age 15-19 years), but decreased in subsequent years below national levels of 6.5% (aged 15-49 years) at 4.8% for females aged 15-24 year old and at 2.3% for males aged 15-24 years [6]. Prevalence of other types of STIs (not HIV) are higher, varying from about 5% among 10-14 years old adolescents (4% for boys and 6% for girls and) to around 12% among 20-24 years old (10% among boys; 16% among girls) [4]. In 2006, teenage pregnancy in Uganda was estimated at 25% [3]. Of the approximately 117,000 unsafe abortions which occur annually, 55% are performed on 17-20 year old girls, with serious complications (23%) and even death (2%) occurring as a consequence. Complications of pregnancy, (often illegal) abortion, and childbirth are the leading causes of disability and death among Ugandan girls aged 15 to 19 [2].

Coerced sexual intercourse is also a major problem for many young Ugandans, both girls and boys [5,8,9]. Due to economic and socio-cultural factors, young people engage in transactional and intergenerational sex. Transactional sex refers to a situation in which an older man (or woman) gives money or gifts to a younger woman (or man), who makes it clear that she engages in sex only in exchange for payment, a ride in men's cars, good marks in school, employment or gifts [5]. It is estimated that approximately 30% of 15 to 24 years old Ugandan females are involved in such transactional relationships with men who are at least 10 years older [5]. Moreover, 16% of the young women and 18% of the young men (age 15-19) are reported to have been engaged in giving or receiving money, gifts, or favours in exchange for sex in the past 12 months [3]. However, girls who receive gifts and money from their boyfriends, and the other way around, for having sex, is also often considered part of normal dating behaviour among Ugandan adolescents [5].

These figures and data show that promoting safe sex behaviour (including strengthening personal decision making skills) among Ugandan adolescents is important. The important role of school-based comprehensive sex education programmes, especially in developing countries, to increase the delay of sexual debut, secondary delay and safe sex behaviour, has been demonstrated in different studies and reviews [10-12].

The present study aims to assess the effects of a comprehensive sex education programme in Uganda, called *The World Starts With Me *(WSWM). It is one of the few large-scale evaluation studies of a school-based comprehensive sex education programme conducted in a Sub-Sahara African setting [1,11].

### The Intervention: The World Starts With Me

In 2002 and 2003, the World Population Foundation (WPF) and Butterfly Works (both Dutch NGO's), together with SchoolNet Uganda (SNU; Ugandan NGO), developed the comprehensive sex education programme *The World Starts With Me *(WSWM). It is a low-tech, computer-based, interactive sex education programme aimed at secondary school students (age 12-19). WSWM was developed by drawing from a combination of (evidence-based) approaches in sex education (e.g. rights-based, health promotion, behaviour change, and adolescent developmental approaches), aiming to empower and support young people in making their own, informed decisions about sex. The experience of past programmes indicates that young people need programmes that are accessible, non-judgemental, and responsive to what young people want [13,14]. WSWM is based on the same principles, combining the development of computer skills and creative expression, and at the same time focusing on sexual and reproductive health and rights. This combination empowers young people not only to obtain the necessary knowledge to develop appropriate attitudes and learn healthy and responsible behaviour and life skills, but also to develop their creative and IT skills in preparation for job opportunities.

The programme uses virtual peer educators, David and Rose, who are the main sources of knowledge and who guide secondary school students in their learning process. The lessons usually start with a theme-based warming up activity, followed by a presentation by Rose and David. The next step is often a game (such as the 'body change game', the 'personality game', the 'who is responsible' game or the 'safe sex quiz'), which serves to help students internalize information and explore opinions. The main part of most lessons is the assignment, for example creating a storyboard, an art work or conduct a role-play addressing the topic of that specific lesson.

WSWM was designed for a setting, like in Uganda, where the computers used in schools tend to be basic and where four to five students have to share one computer. However, as many schools do not have (enough) computers, WSWM also allows students to read the information and to do the assignments and exercises without the computer, using hard copy manuals. This way, the same learning objectives can be met as when WSWM is followed using a computer, although the extra incentive of developing computer skills is lost. WSWM is carried out in English, which is the official language of Uganda.

The role of the teachers is to facilitate the process of learning, coach students to explore opinions, and practise skills by using role play, creating story boards, art work and other (digital) means. Teachers are required to guide students in a non-judgmental way, acknowledging the rights of young people to gain information and to make their own decisions when it comes to their sexuality. To facilitate teachers in this new role, they receive a training lasting 5 to 6 days prior to implementing WSWM in their schools.

The programme consists of 14 lessons, divided into four sections (see Table 1 for an overview of the WSWM lessons). The first section aims to build self-esteem and personal decision-making, gaining insights into a person's identity and sexual development. Personal values and norms are addressed as they form a basis for learning to make personal decisions. In the second section, the role of the social environment (e.g. peers, family, close friends, teachers, and media), gender equity and sexual and reproductive rights are addressed, in order to teach young people to cope with social influences on their own decision making. The third section addresses sexuality as something beautiful, as a vital source for life, but also addresses sexual health problems and the life skills necessary to know how to avoid or deal with them. The fourth section focuses on applying lessons learned about goal setting regarding the students' future and on making booklets for use in peer education. The curriculum closes with an exhibition, at which young people show their parents and the community the results of the class efforts, such as slogans, posters and action plans.

**Table 1:** Overview of "The World Starts With Me" Curriculum

Lesson		Topic
*Section 1: Self-esteem and decision making*		

1	The World Starts With Me	Building self-esteem, insight into own sexual development
2	Emotional Ups and Downs	Exploring processes of autonomy and own norms & values
3	Is Your Body Changing Too?	Body changes

*Section 2: The social environment*		

4	Friends and Relationships	Focus on social environment: Own social
5	Boys and Girls, Men and Women	relationships, own value system, cultural &
6	Fight for your Rights!	political influences, reorienting gender roles & human rights

*Section 3: Sexuality*		

7	Sexuality and Love	Addressing potential of sexuality
8	Pregnancy: 4 Girls and 4 Boys!	Pregnancy and the impact for girls and boys
9	Protect Yourself: STI's; HIV/AIDS	Strengthening personal decision making
10	HIV/AIDS: U have a role 2 play 2	Learning to respect decisions of a partner
11	Love Shouldn't Hurt	Addressing stigma, sexual harassment/abuse

*Section 4: Goal setting*		

12	Your Future, Dreams and Plans	Goal setting and planning for the future
13	My Top Tips Peer Book	Learning how to share goals & plans with
14	The Exhibition	peers and community

The theoretical foundation of WSWM stems largely from two psychological theories of individual behaviour change: the theory of planned behaviour (TPB) [15,16] and the health belief model (HBM) [17]. According to the TPB, (sexual) behaviour is determined by the *intention *to perform a certain behaviour, which in turn is influenced by *attitudes, subjective norms *and *perceived behavioural control *(or *self-efficacy)*. An *attitude *is defined as a person's disposition to respond favourably or unfavourably towards certain behaviour. *Subjective norms *are functions of beliefs that specific, important individuals or groups (e.g. friends, parents, girlfriend, or husband) approve or disapprove of certain behaviour. The *perceived behavioural control *refers to a person's conviction regarding whether or not he has the required skills and resources (power) to perform the behaviour [15,16].

From the HBM [17], the constructs of *perceived susceptibility *and *perceived severity *were used as a basis for the development of the WSWM. *Perceived susceptibility *(or *perceived personal risk*) refers to a person's subjective perception of the risk of contracting a particular condition or illness. *Perceived severity *refers to a person's feelings concerning the seriousness of contracting an illness.

WSWM was first introduced in 30 secondary schools in Uganda in September 2003 as a club-based, extra-curricular programme. In subsequent years, the programme has been introduced in more than 150 schools throughout the country, reaching approximately 15.500 secondary school students in 2009. Since the start, 422 secondary school teachers and 120 peer educators have been trained [18]. It was intended that WSWM would be implemented over a 6-month period in each school. As WSWM is a club-based, extra-curricular programme, the students follow the programme on a voluntary basis, during hours outside the normal curriculum.

### The present study

The present study examines the effectiveness of the WSWM programme in Uganda. It was hypothesized that, following the intervention, students in the intervention group would score significantly better than students in the comparison groups on the main socio-cognitive determinants (knowledge, beliefs, attitudes, perceived social norms, self-efficacy, risk perception and intention) of safe sex behaviour (delaying sexual intercourse; condom use and non-coercive sex).

## Methods

### Study design and sample

The evaluation had a quasi-experimental design, including pre- and post-test, intervention and comparison groups, and was conducted using a self-administered questionnaire. As the intervention schools were already selected, based on the schedule of implementation of the non-governmental organisation SchoolNet Uganda (SNU), a randomized control trial could not be used. For each intervention school, a matching comparison school was selected on the basis of (1) type of school (boarding; day; mixed), (2) boys/girls/mixed school, and (3) geographical area (rural/urban). The comparison schools were promised that they would receive the WSWM programme next year if they were interested (waiting-list control group). The pre-test measurement took place in March 2008, just prior to the start of WSWM in the intervention schools (April 2008). The post-test measurement took place immediately following the end of the intervention period (September/October 2008).

A total of 48 schools (24 intervention and 24 comparison schools) were selected, equally distributed throughout Uganda. To ensure an equal distribution of gender and age, the (head) teachers were asked to select a group of 50 students on the basis of gender and age. The response rate was 83% (*N *= 1986) for the pre-test (T0) and 67% (*N *= 1609) for the post-test (T1). Unavailability for post-test was primarily due to absenteeism, transfer to other schools and drop out. Data from two respondents at post-test were removed, because they had not completed the pre-test survey. Data from three intervention schools (in total 122 respondents) were also removed as these schools had finished only up to lesson 7 of the programme at post-test (two schools were at lesson 4, one school at lesson 7). The respondents of all comparison schools remained included in both pre-test and post-test. Outcome analysis was carried out with a final dataset comprising 1864 respondents at pre-test (853 intervention group; 1011 comparison group) and 1519 respondents at post-test (723 intervention group; 796 comparison group).

Drop out analysis, using one way ANOVA, revealed that those respondents who dropped out after pre-test (*N *= 345), compared with those who were present at both pre-test and post-test, were older (*M *= 16.29*, SD *= 1.82 vs. *M *= 16.04*, SD *= 1.90; *F *(1, 1743) = 4,63, *p *<.05) and scored higher on *performance behaviour of condom use *(*M *= 1.69; *SD *= 1.13 vs. *M *= 1.53; *SD *= 1.04; *F (*1, 1722) = 4,00 *p *<.05). Furthermore, they scored lower on both *beliefs about causes of HIV *(*M *= 3.46; *SD *= .99 vs. *M *= 3.58; *SD *= .97 *F (*1, 1428) = 4,88 *p *< .05) and on *attitudes towards delaying sexual intercourse *(*M *= 3.97; *SD *= 1.06 vs. *M *= 4.13; *SD *= .93 *F (*1, 1687) = 7,84 *p *< .01). A chi-square analysis revealed that those respondents who dropped out were more often boys than girls (53.3% vs. 46.7%), χ^2 ^(1, 1745) *= *10.8, *p *< .001).

### Procedure

Research assistants (first year undergraduate students from the Makerere University in Kampala, Uganda) were selected by SNU and trained over the course of three days by two researchers, one from Uganda and one from the Netherlands. They were given an explanation of the objectives of the study, the objectives and content of the WSWM programme, the structure and rationale of the questionnaire, and the terminology (e.g. "petting & fondling", "other sexual activities", "masturbation") and concepts (e.g., Sexual and Reproductive Health and Rights) involved. They were trained in responding to questions that students might have (e.g., what is meant by "mutual masturbation"?). The research assistants also received instruction on how the questionnaires should be administered for linking purposes; how to instruct the teachers and the students on filling in the questionnaire, and how to create a safe, quiet and anonymous environment in which students could complete the questionnaire.

Students completed the questionnaire in a classroom supervised by the trained research assistants. The research assistants explained to the class that confidentiality was assured by the fact that only the researchers had access to their questionnaires, that no names would be attached to the questionnaires, and that not one single questionnaire would be discussed with the school staff or any other person. Students received a list of definitions with terminology used, like "anal sexual intercourse =....". They also received a referral list with nearby counselling services. After the post-test, the students received a sheet with the main results from the pre-test survey and with correct information on sexual health issues. Participating schools received compensation (on average $50 per school) for costs made to make the evaluation possible. Students received a drink and a snack after completing the questionnaire.

### Ethical clearance

Ethical clearance was granted by the Ethical Committee Psychology (ECP) of Maastricht University in the Netherlands. Each of the respondents signed an informed consent form.

### Study instruments and Measures

A pre-test semi-structured questionnaire was developed to collect data on the socio- demographic characteristics of the respondents, as well as on outcome variables that were central to the WSWM programme. Items and scales were based on a questionnaire previously used in Tanzania and South Africa (SATZ Project) [19]. The questionnaire was pretested among secondary school students in Kampala, Uganda, after which some minor changes were made. The questionnaire had a boys- and girls-version, differing only in the wording of the questions (i.e., questions related to pregnancy, boyfriend vs. girlfriend). The same questionnaire was used at post-test.

Items were scored either on a 5 point Likert scale, ranging from 1= lowest score to 5 = highest score, or on a binary scale ("no" or "yes"). The reliability of the scales was assessed using Cronbach's alpha in cases where the scale consisted of a minimum of 3 items. For two items constructs, Pearson correlation coefficient was used, with a significant correlation level of *p *< .01. The following constructs were measured in the questionnaire:

*Socio-demographic profile of respondents: *Questions on gender, age, religion and sexual experience (having had sexual intercourse or not) were included in the questionnaire and measured using appropriate scales.

*Knowledge concerning risky sexual behaviour leading to STI, HIV or pregnancy*: Students were asked to respond to seven statements testing knowledge about which sexual behaviour could lead (or not lead) to (1) pregnancy, (2) STIs and (3) HIV (e.g., "deep kissing may lead to STI; no vs. yes). After dummy coding the scores to indicate incorrect (0) and correct answers (1), a factor analysis using orthogonal rotation (varimax), revealed five subscales: two scales were found measuring knowledge of the causes of STIs: knowledge about safe sexual behaviour not leading to STI (deep kissing; fondling; petting lead to STI; *α *= .67) and knowledge about unsafe sexual behaviour and possible risk of STI (oral sex; vaginal sex; anal sex lead to STI; *α *= .57). One scale was found measuring knowledge about causes of HIV infection (deep kissing; fondling; petting lead to HIV; *α *= .58) and two scales were found measuring knowledge about (non) causes of pregnancy: (1) deep kissing; fondling; petting & mutual masturbation cannot lead to pregnancy *(α *= .58) and (2) having oral sex and having anal sex cannot lead to pregnancy (*r *= .18; *p *< .01).

*Beliefs concerning pregnancy, STIs and HIV: *Three two-item scales were used to measure respondents' beliefs about safe sex behaviour. Beliefs concerning pregnancy (e.g., "A girl cannot get pregnant the first time she has sex"; *r *= .44*; p *< .01), *beliefs concerning *HIV (e.g., "The HIV virus can be transmitted by mosquito bites"; *r *= .16*; p *< .01), and beliefs concerning STIs (e.g. "Anal sexual intercourse is a safe way to protect oneself from sexually transmitted infections", *r *= .22; *p *< .01 ).

*Risk perception *was measured by three different constructs: two items measuring risk perception (i.e. susceptibility and severity) regarding getting pregnant (e.g., "If I have vaginal sexual intercourse without a condom or another contraceptive, this may lead to pregnancy"; "If I (my lover) become(s) pregnant, I will not be able to fulfil my dreams"; *r *= .17; *p <*.01); 2 similar items related to risk perception regarding HIV infection *(r *= .22; *p *< .01), and 2 similar items related to risk perception regarding getting a STI (*r *= .20; *p *< .01).

*Delay of sexual intercourse. Attitude towards delaying sexual intercourse *was measured with two items: "It is better that young people my age, who are in a steady relationship, postpone sexual intercourse until they are older" and "Young people should not engage in sex until they are married" (*r *= .29; *p *< .01); *perceived social norm about delay of sexual intercourse *was measured with a single item: "My friends believe that people my age should postpone sexual intercourse until they are older". *Self-efficacy towards delaying sexual intercourse *was measured with 2 items: "For me, waiting with sexual intercourse until I am older is difficult" and "I am confident that I can wait to have sexual intercourse until I am older" (*r *= .45; *p *< .01). *Intentions to delay sexual intercourse *were measured by two single items: "Do you think you will wait with sexual intercourse until you are older?" (5 point scale; 1 = very unlikely and 5 = very likely) and "Do you plan to abstain from sexual intercourse until later?" (0 = no; 1 = yes).

*Condom use: *Condom use was measured with four items (e.g. "How often have you obtained a condom in the past 6 months?" and "In the past 6 months, did you use a condom when having sex?"(*α *= .84). *Attitudes towards condom use *were measured by two items, e.g. "using a condom is wise" (*r *= .34; *p *< .01*)*. Students were also asked whether they thought using a condom was pleasant. *Perceived social norm regarding condom use *was measured by one item: "My friends think that people my age should use a condom when having sexual intercourse". *Self-efficacy for condom use *was measured by two items: "For me, using a condom every time I have sexual intercourse is difficult" and "I am sure that I can use a condom every time I have sexual intercourse" (*r *= .32; *p *< .01.) *Intention to use condoms *was measured by two single items (e.g. "Do you think you and your (future) lover will use a condom when you will have sexual intercourse?").

*Non-coercive sex: *For "non-coercive sex", *past behaviour regarding avoiding and escaping risky situations *was measured with two items ("How often in the last 6 months did you avoid a situation in which you would have run the risk of unwanted sex?" and "How often in the last 6 months did you find yourself in a situation in which unwanted sex could have occurred and you managed to get out of it?"; *r *= .54; *p *< .01). A*ttitudes towards sexual coercion and force *were measured with three items (e.g. "When a boy is sexually excited and wants sex, his lover is allowed to refuse him"). *Self-efficacy in dealing with situations where unwanted sex could happen *was measured with six items (e.g. "Refusing a lover who pressures me to have sexual intercourse is difficult"; *α *= .70). *Intention to deal with unwanted sex and force *was measured with two items ("Do you think that in future you will avoid situations in which unwanted sex could happen?", and "Imagine you are in a situation with a lover and you don't want to have sex. Do you think you will refuse him/her?"; *r *= .34; *p *< .01).

### Data analysis

The questionnaire data were coded, validated and cleaned. Data were analysed with SPSS 17.0. Frequency analysis was conducted to describe the demographic characteristics of the study sample at pre-test and one-way ANOVA's were carried out to determine possible statistically significant differences between the intervention and comparison groups at pre-test. To assess intervention effects, a linear mixed model procedure was used. Due to the fact that students belonged to different schools (hierarchical structure), the independence assumption is violated [20]. The Mixed Model procedure accounts for the hierarchical structure of the data and consequently tests the effects of intervention on the dependent variables for the random influence of school membership. Due to the non-random nature of the distribution of schools, and therefore also participants across the intervention and control conditions, we decided to use analysis of variance (ANOVA) of change from pre-test as the primary method of analysis rather than analysis of covariance (ANCOVA) of the outcome with the pre-test as covariate. In nonrandomized studies of pre-existing groups, ANOVA of change seems to be less biased than ANCOVA [21]. We further included age, gender and control/intervention variables as covariates. We were primarily interested in finding a significant group (intervention vs. control) x time (pre-test vs. post-test) interaction effect, which would suggest that the intervention had an effect on the outcome variable. In cases where there was a significant time x group interaction effect, we conducted simple effects analyses testing the effect of group at both pre-test and post-test in order to understand the nature of the intervention effect. As the number and size of clusters were high (45 schools (21 intervention schools and 24 comparison schools) with an average of 42 participants per cluster), this same procedure also allowed us to interpret the significance of the effect of intervention on those dependent variables with a binary distribution. The significance level in this study was set at *p *= .05.

## Results

### Socio-demographic Profile of Students

The mean age of the students was 16.1 years (*SD *= 1.87). More than half of the students were girls (55.2%) and 44.8% were boys. The majority of the students indicated that they were Christian (39.5% Protestant, 34.2% Catholic, 13% Pentecostal), 11.5% were Muslim. At pre-test, 36.8% of the boys (*N *= 836), and 20.1% of the girls (*N *= 1028), had engaged in sexual intercourse. More than half of those who were sexually experienced (60.3%), said they had used a condom when they last had sexual intercourse (56.1% of the boys and 67.3% of the girls).

### Effects of WSWM

Table 2 shows an overview of the mean scores (SD) on the dependent variables at pre-test (T0) and post-test (T1) for both intervention and comparison groups, and the F-test statistic for the time x group interaction effect. Below we discuss the significant interaction effects on the different outcome variables.

**Table 2:** Mean items scores or percentages for experimental and control groups at pre- test and post-test

	Intervention		Comparison		
	**Pre-test** ***(N = 853)*** ***M (SD)***	**Post-test** **(*N = 723)*** ***M (SD)***	**Pre-test** ***(N = 1011)*** ***M (SD)***	**Post-test** ***(N = 796)*** ***M (SD)***	***F***

**Knowledge**					

Knowledge about non-causes of STI (petting, fondling, deep kissing)	.37 (.56)	.31 (.37)	.32 (.37)	.28 (.34)	.38
Knowledge about causes of STI (anal sexual intercourse; vaginal sexual intercourse; mutual masturbation)	.47 (.46)	.64 (.43)	.44 (.37)	.58 (.38)	2.20
Knowledge about non-causes of HIV (petting, fondling, deep kissing)	.38 (.55)	.34 (.38)	.27 (.34)	.30 (.34)	6.02*
Knowledge about non-causes of pregnancy (petting, fondling, deep kissing)	.93 (.45)	.89 (.34)	.90 (.20)	.90 (.21)	.16
Knowledge causes of pregnancy (vaginal sexual intercourse; not anal intercourse)	.26 (.44)	.23 (.42)	.22 (.32)	.20 (.31)	.28

**Beliefs**					

Beliefs about causes of pregnancy	3.76 (.99)	4.18 (.89)	3.78 (.98)	4.03 (.89)	11.16**
Beliefs about causes of HIV	3.60 (.96)	3.74 (.98)	3.53 (.97)	3.72 (.93)	.27
Beliefs about causes of STI	3.27 (.96)	3.58 (.89)	3.25 (.96)	3.51 (.87)	.67

**Risk perception (perceived risk and perceived severity)**					

Risk perception towards HIV	3.92 (.94)	3.90 (.93)	3.89 (.94)	3.95 (.90)	1.07
Risk perception towards STI	3.45 (1.00)	3.55 (.95)	3.49 (.97)	3.55 (.94)	.73
Risk perception towards pregnancy	4.00 (.90)	4.04 (.88)	3.94 (.89)	4.02 (.87)	.21

**Delay**					

Attitudes	4.10 (.97)	4.22 (.94)	4.11 (.95)	4.16 (.97)	1.51
Perceived social norm (single item)	3.59 (1.37)	3.89 (1.24)	3.69 (1.33)	3.78 (1.29)	7.15**
Self-efficacy	4.02 (1.03)	4.17 (.97)	3.94 (1.04)	4.12 (.98)	.03
Intention-1	4.01 (1.27)	4.16 (1.15)	3.93 (1.29)	3.94 (1.26)	4.11*
Intention-2	.91 (.28)	.92 (.28)	.92 (.28)	.87 (.34)	6.98**

**Condom use**					

Attitude (wise to use a condom)	3.78 (1.09)	3.82 (1.04)	3.81 (1.07)	3.74 (1.12)	4.56*
Attitude (pleasant to use a condom)	3.52 (1.29)	3.27 (1.16)	3.54 (1.29)	3.37 (1.22)	1.02
Perceived social norm towards condom use	3.79 (1.21)	3.68 (1.24)	3.86 (1.23)	3.80 (1.20)	.26
Self-efficacy towards condom use	3.31 (1.05)	3.37 (1.01)	3.34 (1.04)	3.27 (1.01)	5.09*
Performance behaviour (buy, carry a condom etc.)	1.94 (1.14)	2.14 (1.11)	2.23 (1.25)	2.53 (1.22)	.46
Intention to use a condom	3.70 (1.35)	3.79 (1.27)	3.82 (1.26)	3.75 (1.27)	3.96*

**Non-coercive sex**					

Performance behaviour to escape and avoid situations where unwanted sex could happen	2.24 (1.33)	2.21 (1.27)	2.28 (1.35)	2.25 (1.30)	.000
Attitudes towards using force for getting sex	3.65 (1.04)	3.78 (.97)	3.64 (1.06)	3.69 (1.04)	1.82
Self-efficacy in dealing with coercive sex	3.74 (.82)	3.96 (.79)	3.76 (.77)	3.88 (.76)	5.51*
Intention to deal with coercive sex	3.93 (1.01)	4.02 (.91)	3.95 (.96)	3.93 (.99)	2.69

* p <.05; ** P <.01 (two-tailed)

*Knowledge about risky sexual behaviour leading to STI, HIV or pregnancy: *Multilevel analysis revealed a reversed effect of intervention on *knowledge scores relating to non-causes of HIV *(petting, fondling and deep kissing). At pre-test, a significant difference between groups was found, *F *(1, 1182) = 18.27, *p *< .000), with the intervention group scoring significantly higher on knowledge of non-causes of HIV than the comparison group. At post-test, students who followed WSWM had, on average, lower knowledge scores as compared to pre-test, whereas students from the comparison group improved their knowledge at post-test as compared to pre-test. However, the intervention group still scored significantly higher at post-test than the comparison group*, F *(1, 1257) = 4.22, *p *< .05). For the other knowledge scales, no significant effects of intervention were found.

*Beliefs about STI, HIV and pregnancy: *Students from the intervention schools scored significantly better than the comparison students at post-test as compared to pre-test when it came to *wrong beliefs concerning pregnancy*, like "a girl cannot get pregnant the first time she has sexual intercourse". At pre-test, there was no significant difference between intervention and comparison groups when it came to wrong beliefs concerning pregnancy, *F *(1, 1810) = 1.12, *p *= .289*)*. However, at post-test, the intervention group scored significantly higher than the comparison group, *F *(1, 1501) = 21.98, *p *< .001. No significant effects were found for beliefs about STIs, or for beliefs about HIV.

*Risk perception: *No significant effects were found on risk perception.

*Delaying sexual intercourse: *We found an interaction effect for intervention over time for *perceived social *norm. At pre-test, there was no significant difference (*F *(1, 1745) = .13, *p *= 1.30) between intervention and comparison groups. At post-test, both intervention and comparison groups agreed significantly more with the statement, "My friends believe that people my age should postpone sexual intercourse until they are older", as compared to pre-test, but this change was only significant for the intervention group. Furthermore, students who had followed WSWM were more convinced at post-test as compared to pre-test that they would *wait with sexual intercourse until they were older *(condition x time interaction effect), (*F *(1, 1385) = 11.18, *p *= .001), whereas there was no significant increase in agreement with this statement for the comparison group at post-test as compared to pre-test. At pre-test, there was no difference between intervention and comparison groups (*F *(1, 1655) = 1.01, *p *= 3.15. The students from the intervention group also held a stronger *intention to delay sexual intercourse *at post-test*, F *(1, 1257) = 7.22, *p *= .007, again with no significant difference between the two groups at pre-test, (*F *(1, 1695) = .01, *p *= .937). No significant effects were found for *attitudes towards delaying sexual intercourse*, nor on self-efficacy *towards delaying sexual intercourse*.

*Condom use: *The analysis revealed a positive effect on *attitude towards using condoms ("It is wise to use a condom" and "everybody should use one")*. At pre-test, there was no significant difference between intervention and comparison groups, *F *(1, 1623) = .34, *p *= .853. The difference at post-test was marginally significant, *F *(1, 1319) = 3.27, *p *= .07, with an increase in positive attitude towards condom use among the intervention group students and a decrease in positive attitude among the comparison group students. After having followed WSWM, the intervention group students also scored significantly higher on *self-efficacy using a condom *than the comparison group, but this effect was primarily due to a marginally higher mean score on self-efficacy among the intervention group students at post-test as compared to pre-test, and a decrease in the mean score on self-efficacy among the comparison group students. At both pre-test (*F *(1, 1747) *= *2.12, *p *= .146) as well as post-test (*F *(1, 1415) *= *1.16, *p *= .282), there were no significant differences between intervention and comparison groups. Finally, the analysis showed an effect of intervention on *the intention to use condoms*. At pre-test, a significant difference was found between intervention and comparison groups, *F *(1, 1555) = 4.59, *p *= .032, with the intervention group being less inclined to use a condom than the comparison group. At post-test, students who followed WSWM showed an increase in intention to use a condom, whereas students from the comparison group had on average lower scores on intention to use a condom as compared to pre-test. Although the difference at post-test between the mean scores for intention to use a condom between the intervention and the comparison groups was not significant (*F *(1, 1275 = .749, *p *= .387), the time x group interaction was. This means that the intervention was relatively effective in increasing the intention to use a condom. No significant effects were found for *past performance behaviour*, or for *perceived social norm towards condom use*.

*Non-coercive sex: *The analysis showed a time x group interaction effect for *self-efficacy in dealing with sexual coercion*. At pre-test, there was no significant difference between intervention and comparison groups, *F *(1, 1817) = .168, *p *= .682. At post-test, both intervention group students and comparison students were more confident that they could deal with situations where sexual pressure and force would be used as compared to pre-test, but the increase in the mean score was significantly higher among the intervention group than in the comparison group, *F *(1, 1467) = 7.73, *p *=.006). No significant effects were found for *past performance behaviour regarding avoiding and escaping risky situations*, for *attitudes towards sexual coercion*, or for *intention to deal with unwanted sex and force*.

### Completeness and Fidelity of implementation: additional analyses

As mentioned before, not all schools had implemented the 14 lessons at post-test (T1). The three intervention schools which had only implemented the first seven lessons (1-7) were deleted from this effect study. In order to determine the role of implementation rate (*completeness*), we included those schools that were originally dropped, back into the analyses. We then compared the scores of learners from these schools with learners in the intervention schools in the original analyses that implemented at least lesson 1 to 10 (21 intervention schools, 853 students), and those in the comparison schools, again using a multilevel approach to the analyses. This analysis revealed that all of the above mentioned significant effects disappeared for the schools that implemented less than 50% of the lessons. However, unexpectedly, we found two reversed effects for the schools which implemented no more than the first seven lessons. One on the *beliefs towards HIV*, where both the students in the comparison group as well as the intervention group scored lower at post-test (T1) than on pre-test (T0), but the intervention students significantly lower than the comparison group, *F *(1, 345)*= *4.07, *p *< .05. Another reversed effect appeared for *perceived social norm towards delaying sexual intercourse*, *F *(1, 363) *= *5.70, *p *< .05. Students in the comparison group agreed significantly more with the statement, "My friends believe that people my age should postpone sexual intercourse until they are older" than students from the schools that implemented only lesson 1-7.

We also explored the possible effects of the differences in the fidelity of programme implementation. Based on the results from a process evaluation conducted among 16 (out of the 21) intervention schools, we were able to distinguish between those schools that implemented the programme according to the manual (= full fidelity) and those schools that implemented the programme not totally according to the manual (= partial fidelity). As there were not enough schools which used computers for delivering the programme, we did not distinguish on the basis of computer use. All teachers, whether they would run the programme using computers or not, implemented the programme by using the WSWM manual. Three questions answered by teachers were used: (1) "Did you teach the lessons in the order that is stated in the WSWM manual" (yes = fully = 1; no = partial = 0); (2) "Of all the assignments/exercises that you taught, did you conduct them in the way they were described in the teacher manual (following the exact description)?" (Yes totally = fully = 1; Yes, partially; not sure; not really; not at all = partial = 0) and (3) "I have closely followed the WSWM manual when conducting the lessons" (Totally agree; agree = fully = 1; neither agree nor disagree; disagree and totally disagree = partial = 0).

Full fidelity schools (8 intervention schools; *N *= 344*) *were compared with partial fidelity schools (8 intervention schools; *N *= 325*) *in a multilevel mixed model design, with gender, age and pre-test measures as covariates. This follow-up analysis revealed that the partial fidelity group scored significantly better on *beliefs concerning pregnancy *and on *knowledge concerning non-causes of pregnancy*. At pre-test, the partial fidelity group scored lower than the full fidelity group concerning *beliefs towards pregnancy*, but not significantly lower. Both partial and full fidelity groups showed an increased mean score at post-test, but the increase among the partial fidelity group was significantly higher than the full fidelity group, *F *(1, 587) *= *8.53, *p *< .01. Also for *knowledge concerning non-causes of pregnancy*, the partial fidelity group scored lower on average at pre-test than the full fidelity group. However, students in the partial fidelity group improved their knowledge significantly more than the full fidelity group at post-test*, F *(1, 514) *= *8.51, *p *< .01.

## Discussion

The present study is one of the few large-scale evaluations of a school-based sex education programme in Sub-Saharan Africa [1,11]. The findings of this study show that students from the intervention group which completed at least the first 10 out of the 14 lessons were significantly better able to detect wrong beliefs on how to prevent pregnancy; had a significantly higher score on perceived social norm towards delaying sexual intercourse; were more convinced that they would delay until they were older, and also scored higher on the actual intention to delay sexual intercourse. Students from the intervention group held a significantly more positive attitude towards condom use, and were more convinced that they would be able and self-confident enough to use a condom in future when having sexual intercourse. They also had a significant higher intention to use a condom in future. Finally, the intervention group scored significantly higher than the comparison group on self-efficacy in dealing with sexual violence (pressure and force for unwanted sex). The comparison group had significantly improved their knowledge on the (non) causes of HIV from pre- to post-test compared to the intervention group. However, the intervention group had an overall better knowledge on all issues than the comparison group at both pre-test as post-test, except for the non-causes of pregnancy at post-test. The absence of some effects could be explained by the fact that both intervention as well as comparison groups already had a high score at pre-test. Attitude towards delay, for instance, was already positive at pre-test for both intervention and comparison groups (see Table 2).

The determinants on which the intervention had a significant positive effect mainly stem from the TPB-model. Risk perception (perceived severity and perceived susceptibility), a construct stemming from the HBM, did not significantly change due to the intervention. This is in line with previous research, which concluded that the TPB has received considerably more support from research for its predictive power of safe sex behaviour than the HBM [22-25]. On the other hand, both intervention and comparison groups already held high scores regarding risk perception at pre-test (see Table 2). This is in line with results from the 2004 national survey among adolescents in Uganda [4].

In general, the results on intervention effects are in line with results from previous studies conducted in a (Sub-Saharan) African context [1,8,12]. Systematic reviews of studies on school-based sexual health interventions to prevent unintended pregnancies, STIs and HIV in (Sub-Saharan) Africa have shown that knowledge and attitudes regarding STIs, HIV and AIDS can be changed positively, provided that the programme has been carefully designed to suit the (Sub-Saharan) African context. Changing intentions regarding safe sex, especially intention to use condoms, is more difficult. Results from different studies show that actual condom use requires more than knowledge, positive attitudes, and beliefs related to condom use, especially in a Sub-Saharan context where the social norms dictate *no sex until marriage *[12], a notion supported in Uganda by law and religion [26]. Self-efficacy and skills (for example being able to openly discuss and negotiate condom use), especially for girls, need to be addressed explicitly in interventions [11,27]. It is important not only to provide information, but also to take the wider (community and school based) context into account [12,28]. Questions like, "Do students have easy access to condoms if needed?"; "Can they talk with some adult confidentially about sexual issues?", and "What happens if a girl gets pregnant whilst still at school?" raise important socio-cultural issues that should be taken into account.

As full implementation of interventions cannot automatically be assumed, especially not in a context where time, financial and other capacity barriers interfere with full implementation [12,26,29,30], we also looked at whether all lessons were implemented at post-test (*completeness of implementation*) and whether teachers implemented the programme according to the manual or not (*fidelity of implementation*). Follow-up analysis between those schools which implemented at least 10 out of the 14 lessons at post-test and schools which did not complete more than the first seven lessons at post-test (*completeness of implementation) *revealed that in the latter group, all positive effects disappeared. Two reversed effects appeared, one for *perceived social norm towards delaying sexual intercourse *and one for *beliefs towards HIV*. The results from the follow-up comparative analysis based on *fidelity of implementation *revealed that the partial-fidelity group scored significantly better on *beliefs towards pregnancy *and on *knowledge of non-causes of pregnancy *than the full-fidelity group. In other words, whether a teacher strictly followed the instruction manual or not, did not have a big impact on the effectiveness of WSWM. On the contrary, the limited effects found were in favour of the partial-fidelity group. These results suggest that it is important to implement the programme completely and that the programme itself should be flexible enough for teachers to use and adapt it according to the specific context in which they have to implement the programme (i.e. adapting to accommodate specific questions and needs of the students and coping with the limited time and resources available), without having to skip lessons.

WSWM was developed as a computer-based programme, enabling students to follow the lessons relatively autonomously, offering them privacy to explore and discuss sex-related issues among themselves. Although WSWM can also be implemented without computers, by making use of a Student Manual, the use of computers makes it easier for teachers and students to deal with sensitive topics and makes following the programme more attractive and fun. The use of computers also enables young people to learn important computer skills that would be an asset to them in the labour market after graduation. However, in practice, the use of computers for WSWM appeared to be very limited. Process evaluation showed that most schools did not have enough computers. If there were any, they had to be shared by a group of students (sometimes up to 50) at the same time. Also, broken computers and lack of electricity were major implementation problems faced by most of the schools [31]. We were not able to make a sensible distinction on the basis of computer use, as there were practically no schools where the WSWM was implemented by the use of computers in the way it was intended. The fact that WSWM was implemented in most schools without (enough) computers available for the students, could partly explain the limited effects we found, especially when it comes to very sensitive topics like "coercive sex". When planning for a computer-based programme in a context like Uganda, where many schools do not have (enough) computers and/or do not have a secure access to energy, the implementation plan of the programme should contain a school computerisation component.

The role of teachers is to guide and coach the students through the 14 lessons, facilitating the process of learning, coaching students to explore opinions, and practising skills. In order for programmes to be faithfully implemented, it is important that teachers are properly trained and committed to the programme [12]. WSWM Teachers were trained to do this in a 5 to 6-day training course, conducted by Ugandan trainers, who were trained themselves by either Dutch trainers or Ugandan trainers (Train the Trainer concept). The group of trainers that have trained new WSWM teachers in recent years is different to the first group of Dutch trainers. Some teachers expressed their concern about the differences in openness between the WSWM teacher trainers. Issues such as, for example, homosexuality and condom use were said to be approached differently by the different trainers. This suggests that teachers were not trained effectively in this regard. The process of evaluation also revealed that teachers felt that one training course lasting six days was a good start, but did not provide sufficient support to implement WSWM. They particularly indicated the need for more support, for instance by having a team of experts visiting the schools during implementation of the programme, and the need for follow-up training [31].

Many of these problems could be addressed in a more structural way if the programme could be part of the school curriculum [27]. The few recent studies conducted in Sub-Saharan Africa indicate that curriculum-based, adult-led interventions show stronger evidence of effectiveness than non-curriculum based interventions [32].

Most evaluation studies focus on the effects of the intervention on change in (determinants) of sexual behaviour of the target group only. Process evaluations focusing on how the intervention was implemented are often neglected or less widely publicised. This is remarkable, as a successful adoption and implementation of an intervention is crucial for its success and effectiveness. If the implementation of programmes is not assessed, it is difficult to determine why outcomes were or were not achieved and erroneous conclusions may then be drawn stating that a programme is not effective, when it was simply not delivered as planned [26,30]. Specific attention should be paid to the context of intervention implementation [12,29]. This is especially true for school-based sex education programmes implemented in developing countries, in a context of limited time and (financial) resources, shortage of staff and in which teachers are only allowed to promote abstinence, which is not in line with the reality of the students and which does not help them to distinguish between more and less risky behaviour. Involvement of relevant stakeholders like the Ministry of Education, (Head) teachers, SRHR Specialists, and students in the development, implementation and evaluation of an intervention is crucial for its success. Long-term training and on-the-job support of teachers so that they are able to deliver a programme the way it is intended, are important determinants of success. Systematic planning, implementation and evaluation of interventions are much needed to achieve these goals [28,33].

Our study had a non-randomised experimental design. Due to practical considerations, we were not able to randomise our sample. We did, however, have a large sample of intervention schools (24) and were able to find comparable schools (24) for each intervention school, based on geographical area, boys/girls/mixed school and boarding versus day school. The questionnaire was not translated into local languages. As the level of the students' English differed, we realised that some questions may have been difficult to understand for some respondents. We tried to accommodate for this by training the research assistants to give vernacular translations for those students who failed to understand certain words or concepts. Unfortunately, this was not always possible, as some research assistants did not speak the local language. We tried to assure anonymity in responding, by having no names attached to the questionnaires and by instructing the research assistants to create a safe and quiet environment in which the respondents could fill in the questionnaire. We are not sure if this was achieved at all schools in which the students completed the questionnaire. Students had to sit in the classroom, and sometimes the teacher would also be present, despite the request from the research assistants to leave the room. The scales that we used did not always show the required reliability (Cronbach's *α*), which is why we mostly had to rely on two-items and single item constructs. As the study covered a period of only 6 months, and the post-test was conducted immediately at the end of the implementation period of the programme, we did not include change in actual sexual risk behaviour following the programme. Future research should have follow-up measurements at 6 months and 12 months after the intervention period has finished to measure change in actual sexual behaviour.

## Conclusions

The World Starts With Me is a comprehensive sex education programme, which if implemented completely, can affect changes in socio-cognitive determinants of safe sexual behaviour among young adolescents in Uganda. The finding that the effects of intervention disappeared for those schools that did not complete the programme in time, and the fact that schools where the teacher did not implement the programme fully according to the manual were slightly more effective than those schools that did stick to the manual, raise questions about the role of implementation and the context in which implementation takes place. Socio-cultural (e.g. condom use among young people is a taboo, so teachers are reluctant to promote condom use), political (e.g. homosexuality is a criminal offence, so it is impossible to talk about homosexuality as a human right) and economic (e.g. schools do not have a lot of money to spend, so the use of computers is low; time constraints) characteristics of the context might either facilitate or prevent sound implementation of the programme. Although the WSWM programme in itself seems to be effective, it needs to be developed in such a way that it can be easily adapted to suit the implementation context in each specific school. As WSWM is not part of the school curriculum, the programme should be made more flexible in such a way that it enables teachers to make logical adaptations according to time constraints and to address specific problems and issues faced by the students. When planning for a computer-based programme in a context like Uganda, where many schools do not have (enough) computers and/or do not have a secure access to energy, the programme implementation plan should contain a specific section addressing this issue. Apart from the reason of having limited time, it is unknown why some schools only had implemented 50% of the lessons at post-test, whereas other schools did manage to implement the full 14 lessons in time. More research is needed on factors influencing the complete and successful implementation of comprehensive sex education programmes such as WSWM in Uganda.

## Competing interests

The authors declare that they have no competing interests.

## Authors' contributions

LER participated in the design and the overall coordination of the study, conducted the analysis and drafted the manuscript. AERB and RACR participated in the design of the study, checked the analysis and contributed to the writing of the manuscript. JNL participated in the design and the overall coordination of the study. BdH participated in the design of the study and coordinated the data collection and cleaning in Uganda. All these authors read and approved the final draft. HPS acted as a research coordinator and greatly contributed to the development of the research design and instrument, until his death in 2009.

## Pre-publication history

The pre-publication history for this paper can be accessed here:

http://www.biomedcentral.com/1471-2458/11/334/prepub
